# Modulation of feedback processing by social context in social anxiety disorder (SAD)–an event-related potentials (ERPs) study

**DOI:** 10.1038/s41598-019-41268-0

**Published:** 2019-03-18

**Authors:** Rolf Voegler, Jutta Peterburs, Christian Bellebaum, Thomas Straube

**Affiliations:** 1 Institute of Medical Psychology and Systems Neuroscience, University of Münster, Von-Esmarch-Str. 52, 48149 Münster, Germany; 2 Department of Biological Psychology, Institute for Experimental Psychology, Heinrich-Heine-University, Universitätsstraße 1, 40225 Düsseldorf, Germany

## Abstract

The ability to learn from feedback, especially under social scrutiny, is an essential prerequisite for successful interaction with the environment. Patients suffering from social anxiety disorder (SAD) have been proposed to show altered processing of and learning from feedback, especially depending on social context. However, the neural basis and behavioral consequences of altered reinforcement learning in SAD are not clear yet. In the present event-related potentials (ERPs) study, 34 SAD patients and 30 healthy control subjects (HC) performed an adapted version of a probabilistic feedback learning task in two distinct social conditions. In the observation condition, participants were observed by a confederate; in the control condition, they performed the task without being observed. Patients as compared to healthy controls experienced more subjective discomfort under social observation. Moreover, they showed better learning from negative feedback in the control condition, but reduced learning from negative feedback in the observation condition. This effect correlated with reduced differentiation of positive and negative feedback in the time range of the feedback-related negativity (FRN) under high action-feedback contingency. In addition, SAD patients demonstrated increased FRN amplitudes in the first half of the observation condition, in particular to positive feedback. The present results demonstrate that processing of and learning from feedback are altered in SAD, especially under social scrutiny. In particular, it appears that SAD patients do not process positive information adequately on the neural level, which may impair their ability to differentiate between negative and positive outcomes.

## Introduction

Patients suffering from social anxiety disorder (SAD; DSM-V)^1^ are especially concerned about receiving negative feedback from others. In general, SAD patients fear that their actions might be judged as inappropriate or embarrassing. Being under scrutiny, e.g., in performance situations such as when giving a talk or when talking to a superior, often leads to typical physiological symptoms such as blushing, sweating, or trembling and intense feelings of anxiety. Current cognitive models assume that information processing biases in social situations are central features of SAD. Patients exhibit general negative beliefs about themselves and the consequences of their performance as well as exaggerated standards for their own behavior^2,3^. This may also lead to aberrations in cognitive functions^4,5^. In the dominating cognitive behavioral models of SAD, monitoring processes are identified as paramount factors for the development and maintenance of SAD. According to the influential model set forth by Clark and Wells^2^, SAD patients shift their attention to detailed monitoring and observation of their performance and their own physiological reactions whenever they are faced with potentially threatening social situations, such as social observation. External cues are less attended to, potentially prohibiting adequate processing of external information. In contrast, Rapee and Heimberg^3^ assume that monitoring of both internal and external threat cues is increased in SAD, and that these processes may interact. External social information, for example, may trigger increased monitoring of internal processes and vice versa. Along these lines, alterations in performance monitoring are of particular relevance for the understanding of SAD psychopathology, even though the two theories make different predictions about their exact nature. In both accounts, it is conceivable that altered processing of action outcomes (e.g., feedback) in SAD may also link to altered learning from feedback, i.e., behavioral adaption based on feedback information.

The ability to learn from feedback is an essential prerequisite for successful interaction with the environment. It allows us to learn contingencies between actions and outcomes and to anticipate the consequences of a specific behavior. There is initial evidence that several aspects of feedback learning are altered in SAD. Behavioral investigations have demonstrated that social anxiety is associated with impaired memory for positive social feedback^6,7^, i.e., feedback from others about one’s own performance. In addition, behavioral adaptation, reflected in feedback learning, may also be altered in SAD, although this has not been investigated yet in clinical samples. However, recent studies investigating subclinical samples reported that high socially anxious individuals learned better than a control group to avoid negatively reinforced^8^ or ambiguous stimuli^9^ in probabilistic learning tasks. In sum, SAD patients appear to show a bias towards negative information when learning from both social and non-social feedback. Overall performance, however, appears to be unimpaired, suggesting that social anxiety is probably not associated with deficits in reinforcement learning per se^7,8^.

Neurophysiological mechanisms underlying feedback processing and reinforcement learning have been investigated extensively in studies applying electroencephalography (EEG). The most important event-related potential (ERP) components proposed to reflect these processes are the feedback related negativity (FRN) and P3. The FRN is a component of the ERP that is maximal at frontocentral sites approximately 250 ms after the presentation of external feedback^10–12^. The FRN is usually more pronounced after negative feedback and also for unexpected compared to expected feedback^13^. However, recent evidence suggests that the neural response to positive rather than negative feedback may account for most of the variation of the FRN, possibly by a reward positivity that in turn modulates an N2-like component^14–17^. In addition, the FRN is influenced by feedback relevance (e.g., whether feedback is associated with a particular reward/punishment)^18,19^, and the magnitude of the FRN tends to decrease over time, probably due to increased reward expectancy in the course of feedback learning experiments^20,21^. In a more general framework, it has been proposed that the FRN signals the need for adaptive control in uncertain situations, and that this is moderated by anxiety^22^.

The P3 is a positive ERP component peaking between 300 and 600 ms after stimulus presentation that is most prominent at centroparietal sites^23,24^. One of the most influential theoretical accounts argues that the P3 reflects updating of the mental representation of a given task, so that motivationally significant events receive more attention, as reflected by a stronger P3^25^.

Only one previous study has examined the neural correlates of feedback processing in SAD. Cao *et al*.^26^ reported that the FRN was larger for positive as compared to negative social feedback in SAD patients, while no such difference emerged for healthy control subjects (HC). The authors assumed that this result might be due to the patients’ distinct expectancy to be socially rejected, rendering positive feedback a more surprising event. For the P3, no difference was found between groups. However, given recent conceptualizations of the FRN as mainly driven by reward-associated positivity, this finding might indicate reduced reward processing in SAD, as also suggested by a recent fMRI study^27^.

Overall, there is a surprising lack of studies investigating the neural basis of feedback processing and feedback learning in SAD. Beyond that, it is unclear how social scrutiny, i.e. establishing a directly anxiety-provoking situation, may affect feedback processing and reinforcement learning in SAD. Such manipulations of social context may be of crucial importance for a deeper understanding of the disorder. In a recent fMRI study, Becker *et al*.^27^ found that under alleged social observation, HC, but not SAD patients, showed increased activity in reward-associated brain regions in response to positive feedback. This finding may indicate that feedback processing is altered in SAD as a function of feedback valence as well as social context.

Furthermore, it is still unclear how ERP measures may relate to behavioral characteristics of feedback learning in HC and patients with SAD. In a study by Frank *et al*.^28^, the degree to which healthy participants learned from negative or positive feedback, i.e., whether they were classified as ‘negative learners’ or ‘positive learners’, was linked to FRN magnitude. Negative learners, i.e., subjects who showed a tendency to learn better from negative as compared to positive feedback, showed a relatively stronger FRN to negative as compared to positive feedback than positive learners. The P3 was unrelated to feedback learning. In contrast, Chase *et al*.^29^ reported that P3 but not FRN amplitudes predicted behavioral adjustment in a probabilistic reversal learning task. It is as yet unclear how (and if) these processes are altered in SAD in general and under social observation in particular.

The purpose of the present study therefore was to explore the neural correlates of feedback processing and their modulation by social context in a sample of patients suffering from SAD and matched HC. Moreover, we aimed to elucidate the relationship between ERP measures and behavioral indices of feedback learning in SAD. To this end, SAD patients and HC performed a probabilistic feedback learning task with varying action-feedback contingencies in two distinct conditions. In the *social observation condition*, participants were observed by a confederate while they performed the task. In the *control condition*, participants performed the task without being observed by a confederate. This modulation of social context allowed us to investigate effects of changes in state anxiety, which have previously been shown to significantly affect processes involved in performance monitoring and feedback processing in SAD^4,27^. In particular, we investigated how FRN and P3 were related to learning, and how they were modulated by social context (observation vs. no observation) and psychopathology (SAD vs. HC). The probabilistic feedback learning paradigm applied here allows for an investigation of several core aspects of feedback processing and feedback learning. Differentiation of feedback valence on the neural level (i.e. positive vs. negative feedback) can be measured by means of FRN amplitude. Basic memory for feedback information is reflected in the overall learning rates over the course of the experiment. The information obtained in a transfer block (please see the Methods section for detailed information) indicates the relevance of positive/negative feedback information for behavioral adaption. In addition, results from the transfer block can be related to electrophysiological data to elucidate how FRN amplitude may relate to overt behavior. Based on previous research^8^, we hypothesized that SAD patients would show better learning from negative feedback, while HC should tend to learn more from positive feedback. This pattern should be more pronounced in the observation condition. With regard to ERP measures, we expected FRN and P3 amplitudes to be augmented in the observation condition in HC. For SAD patients, we tested competing hypotheses. According to the Clark and Wells model^2^, SAD patients should be inclined to focus on internal monitoring processes and therefore show reduced amplitudes to external feedback under social scrutiny. According to the model by Rapee and Heimberg^3^, in contrast, they should focus on both internal and external threat^30^. FRN amplitude should therefore be increased in the observation condition. Due to the sensitivity of the FRN to expectation effects, all effects were hypothesized to be most pronounced under high action feedback contingency.

## Materials and Methods

### Participants

An overview of sociodemographic data is provided in Table 1. A sample of 34 patients with social anxiety disorder (mean age: 24.94 ± 2.80 years; 20 females) and 30 healthy adult volunteers (mean age: 24.47 ± 6.67 years; 20 females) was recruited at the Institute of Medical Psychology and Systems Neuroscience at the University of Muenster, Germany. The two groups were matched according to gender, age, intelligence, and educational attainment. All subjects had normal or corrected-to-normal vision, and all were screened for neurological and mental disorders before participation. Exclusion criteria for all participants included psychotic, substance-related or neurological disorders, in particular a history of seizures or head injury with loss of consciousness, and severe uncontrollable medical conditions potentially influencing neurocognitive function.

**Table 1:** Socio-demographic and clinical characteristics of patients and healthy control subjects.

*Demographics*	Controls (n = 30)	Patients (n = 34)
	Mean (SD)	Mean (SD)
Age (years)	24.47 (±6.69)	24.94 (±2.80)
Gender (male:female)	10:20	14:20
Years of education	12.77 (±0.77)	12.76 (±0.43)
IQ (MWT-B)	114.00 (±13.45)	113.25 (±13.14)
***Clinical characteristics***		
BDI	2.61 (±2.77)	9.88 (±6.51)
LSAS	12.60 (±8.36)	61.15 (±20.33)
SPS	5.10 (±4.81)	28.38 (±11.95)
SIAS	10.20 (±5.96)	44.88 (±13.63)
Discomfort during observation*	2.50 (±2.16)	4.80 (±2.28)

SD = standard deviation. *Discomfort rated on a 9-point Likert scale.

An experienced clinical psychologist (R.V.) assessed the current diagnostic status of patients using the German translation of the Structured Clinical Interview (SCID) for DSM-IV (SKID-I)^31^. None of the participants fulfilled the criteria for diagnosis of a current episode of major depression, obsessive compulsive disorder, general anxiety disorder, eating disorder, psychotic disorder, or substance abuse according to the SCID. Four SAD patients reported previous episodes of major depressive disorder. Nine of the SAD patients were in cognitive behavioral treatment and three received psychopharmacological treatment (two with citalopram, one with venlafaxine). All participants completed the Beck-Depression Inventory (BDI-II)^32^, the Social Phobia Scale (SPS), the Social Interaction Anxiety Scale (SIAS)^33^, and the self-report version of the Liebowitz Social Anxiety Scale (LSAS)^34^. Before starting the experimental procedure, all participants gave written informed consent. The study was conducted in accordance with the guidelines of ethical standards in the Declaration of Helsinki and was approved by the Ethics Committee of the German Psychological Society (Deutsche Gesellschaft für Psychologie, DGPs). All subjects received monetary reimbursement for participation.

### Experimental task

The experimental paradigm was an adapted version^35^ of the probabilistic learning task described by Frank *et al*.^36^. On each trial, subjects were presented with one of three different stimulus pairs (AB, CD, EF) composed by two Japanese Hiragana characters. They were asked to choose one of these characters on every trial using the right or left CTRL key on a standard computer keyboard. They were further instructed to try to learn based on feedback which character was correct and to try to respond accordingly as often as possible in order to maximize payout. Positive feedback was associated with wins of 20ct, while negative feedback was associated with losses of 10ct. Unbeknownst to the participants, feedback was not deterministic, but probabilistic. For stimulus pair AB, choosing stimulus A was associated with positive feedback in 80% of trials, while choosing stimulus B was rewarded in 20% of trials. For stimulus pairs CD and EF, the ratios were 70–30 and 60–40, respectively, rendering AB the easiest pair to learn, while EF was the most difficult.

**Figure 1:**
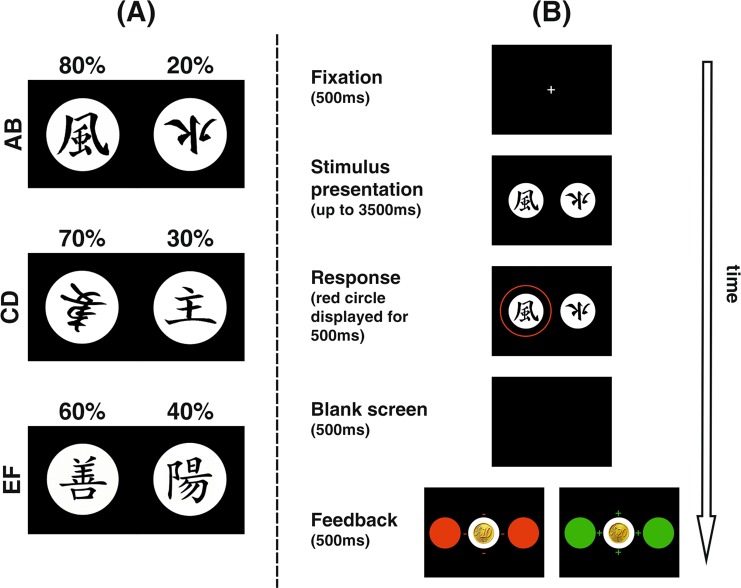
Left panel (A) shows stimulus pairs AB, CD and EF and corresponding probabilities of positive feedback. Right panel (B) illustrates the time course of a single trial in the probabilistic feedback learning task.

Figure 1 illustrates the time course of stimulus presentation in each trial of the task. Each trial started with a fixation cross (presented for 500 ms), before one of the three stimulus pairs was presented. Stimulus pairs were displayed until a response (choice of left or right symbol by button press, indicated by a red circle around the chosen symbol) was recorded, or switched off after a maximum response time window of 3500 ms had elapsed. Subsequently, a black screen appeared for 500 ms before feedback was presented for 500 ms, followed by another blank screen for 500 ms. If subjects failed to respond within the required timeframe, they were instructed to respond faster. The experiment was controlled using Presentation software (Neurobehavioral Systems Inc., Berkeley, CA, USA).

Crucially, all subjects performed two runs of the task in different context conditions (observation/control). Two parallel versions of the experiment with distinct stimulus sets were used to ensure that feedback learning had to begin anew at the beginning of each run. The order of conditions and the assignment of the two different versions of the experiment to the conditions were counterbalanced across participants. Each of the two runs consisted of 4 blocks in each of which every stimulus pair was presented 20 times, amounting to a total of 240 trials per run and 480 trials in total.

Before the observation condition was started, participants were informed that a trained psychologist would observe them during the run. A confederate dressed in a white lab coat then entered the room, turned on a webcam, and subjects were shown a live video feed of themselves seated in front of the computer screen. The experimenter informed the subjects that the trained psychologist would watch them constantly during the next run (both directly and by means of the camera feed), with focus on evaluating their performance in the task. The confederate would take notes on a chart whenever feedback was displayed in order to keep track of their learning progress. The experimenter then started the task and gave general task instructions.

At the end of the last block, a “transfer” block was completed to determine to what extent participants had learned better from positive and negative feedback, during the acquisition phase. To this end, the “best” (A) and “worst” (B) stimuli were paired with stimuli C, D, E, and F to form new stimulus pairs. These were tested on additional 40 trials (20 involving A, 20 involving B). Under these circumstances, choosing A reflects learning from positive feedback, i.e., choosing the symbol with the highest probability of reward over all others, whereas avoiding B reflects learning from negative feedback, i.e. avoidance of the stimulus with the lowest probability of reward. The ratio of ‘choosing A’ vs. ‘avoiding B’ can therefore be used to determine an implicit tendency to learn from positive and/or negative information^8,36,37^.

After completion of the observation run, the confederate briefly thanked the participant and left the room. In the non-observation condition, the camera was (or remained) turned off and the experimenter was seated behind a curtain to avert eye contact, but remained in the room to ensure undisturbed execution of the procedure. The experimenter explicitly informed subjects that they would not be observed during this run.

After each run had been completed, participants were asked to rate their arousal level and feelings of unpleasantness related to positive and negative feedback on Likert scales from 1 to 9. After the observational run, this questionnaire included a question that assessed how uncomfortable subjects had felt while being observed, also on a Likert scale ranging from 1 (not uncomfortable at all) to 9 (extremely uncomfortable).

### Procedure

Before the start of the experimental procedure, all participants were informed that the experiment involved a computerized feedback learning task and that they would be observed by an experienced psychologist during either the first or second run of the task. Afterwards, informed consent was obtained, demographic information was collected, and subjects completed the clinical questionnaires (BDI, LSAS, SPS-SIAS). Then, EEG electrodes were attached and the experimental task was started. Participants were seated in a dimly lit room at a viewing distance of approximately 80 cm from a computer screen. A webcam was placed on top of the computer screen and oriented towards the subject. After the second run, participants completed the IQ test (MWT-B) and were debriefed. The experimental task lasted approximately 50 minutes. Including EEG preparations, IQ testing and questionnaires, the entire session lasted approximately 120 minutes.

### EEG data acquisition, preprocessing and analysis

#### Psychophysiological recordings

EEG was recorded from 64 scalp sites using a BioSemi EEG system with active electrodes, AD-box (BioSemi B.V., Amsterdam, Netherlands), and ActiView software at a sampling rate of 512 Hz. The BioSemi system uses a CMS/DRL feedback loop with two additional electrodes instead of ground and reference (see http://www.biosemi.com/faq/cms&drl.htm). Active Ag-AgCl electrodes were fitted to an elastic cap according to the 10–20 system (FP1, FT9, AF3, F1, F3, F5, F7, FT7, TP9, FC3, FC1, C1, C3, C5, T7, TP7, PO9, CP3, CP1, P1, P3, O9, P7, P9, PO7, PO3, O1, Iz, Oz, POz, Pz, CPz, FPz, FP2, FT10, AF4, AFz, Fz, F2, F4, F6, F8, FT8, TP10, FC4, FC2, FCz, Cz, C2, C4, C6, T8, TP8, PO10, CP4, CP2, P2, P4, O10, P8, P10, PO8, PO4, and O2). Horizontal (HEOG) and vertical eye movements (VEOG) were tracked with additional electrodes attached to the left and right canthi and above and below the right eye, respectively.

EEG-data were analyzed off-line using BrainVision Analyzer 2 software (Brain Products, Munich, Germany) and MATLAB (Mathworks, Natick, Massachusetts, USA). Initially, 0.1 Hz high-pass and 30 Hz low-pass filters were applied to the raw data. Ocular correction was performed based on HEOG and VEOG channels according to the Gratton & Coles algorithm implemented in BrainVision Analyzer 2 software. ERP segments were created ranging from 200 ms before to 600 ms after feedback onset. Baseline correction was performed based on the average signal in the 200 ms directly preceding feedback presentation. Segments containing maximum amplitudes exceeding absolute values of 100 µV or a voltage step of 50 µV were excluded by means of automatic artifact detection. Artifact-free segments were pooled and averaged according to condition (observation/control), valence (positive/negative), contingency (i.e. stimulus pairs AB, CD, EF), and time during the experiment (1^st^ half of each run/2^nd^ half of each run).

The ERP components analyzed for this report included FRN and P3. The FRN was determined as the amplitude difference between the maximum negative peak occurring 200 to 330 ms after feedback onset and the preceding maximum positive peak from 80 ms to 200 ms after feedback onset at electrode FCz. In accordance with previous studies^23,24^, the P3 was defined as mean amplitude in the time window 300 to 450 ms post-feedback at electrode Pz. For correlational analyses, ∆FRN and ∆P3 were calculated as the difference between the FRN/P3 to negative minus the FRN/P3 to positive feedback for stimulus pair AB^28^.

### Statistical analysis

Behavioral and EEG data were analyzed using SPSS (IBM SPSS Statistics 24, IBM Corp., Armonk, New York, USA). Responses in the Frank task were rated as correct if the symbol with higher probability of positive feedback was chosen. Accuracy was analyzed separately for each stimulus pair (AB, CD, EF) by means of repeated-measures analyses of variance (ANOVAs), with condition (observation, control) and time (1^st^ half, 2^nd^ half) as within-subjects factors, and group (SAD, HC) as between-subjects factor. Response times were analyzed with separate repeated-measures ANOVAs for each stimulus pair (AB, CD, EF), with condition (observation, control), time (1^st^ half, 2^nd^ half), and feedback valence (positive, negative) as within-subjects factors, and group (SAD, HC) as between-subjects factor. Response accuracy in the transfer block was analyzed by means of repeated-measures ANOVA with condition (observation, control) and valence (positive, negative) as within-subjects factors, and group (SAD, HC) as between-subjects factor.

Post-run (dis)comfort ratings were analyzed using a repeated-measures ANOVA with condition (observation, control) as within-subjects factor and group (SAD, HC) as between-subjects factor.

In line with analysis of behavioral data, FRN and P3 were analyzed separately for each stimulus pair (AB, CD, EF) by means of separate 2 × 2 × 2 × 2 repeated-measures ANOVAs, with condition (observation, control), time (1^st^ half, 2^nd^ half), and valence (positive, negative feedback) as within-subjects factors, and group (SAD, HC) as between-subjects factor. Time was added as a factor, as already in the analysis of the behavioral data, because previous studies reported a general decrease of FRN amplitudes over the time course of feedback learning experiments, partially related to increasing insight into stimulus-outcome associations^20,21^.

To elucidate the relationship between learning type (positive and negative learning) and ERP measures, results from the transfer learning block were correlated with ∆FRN and ∆P3 separately for SAD and HC. To assess the influence of depressive symptoms on our data, we calculated correlations between BDI and FRN data separately for SAD and HC.

Significance was set to *p* < 0.05. Greenhouse-Geisser correction was applied whenever the assumption of sphericity was violated. Interactions were resolved using lower-ranking ANOVAs and/or post-hoc paired-sample t-tests, with Bonferroni corrected significance levels wherever appropriate.

## Results

### Demographic and clinical data

Demographic and clinical data for SAD patients and HC are provided in Table 1. There were no significant group differences with respect to age (*p* = 0.707), gender (*p* = 0.518), educational attainment (*p* = 0.990), and general intelligence (*p* = 0.825). With regard to clinical characteristics, SAD patients reported a significantly higher number of depressive symptoms than HC, according to BDI scores (*t*(62) = −5.510, *p* < 0.001). They also scored higher on social anxiety measures, as assessed by means of LSAS (*t*(62) = −12.193, *p* < 0.001), SPS (*t*(62) = −9.9974, *p* < 0.001) and SIAS (*t*(62) = −12.877, *p* < 0.001).

### Behavioral data - accuracy

**Figure 2:**
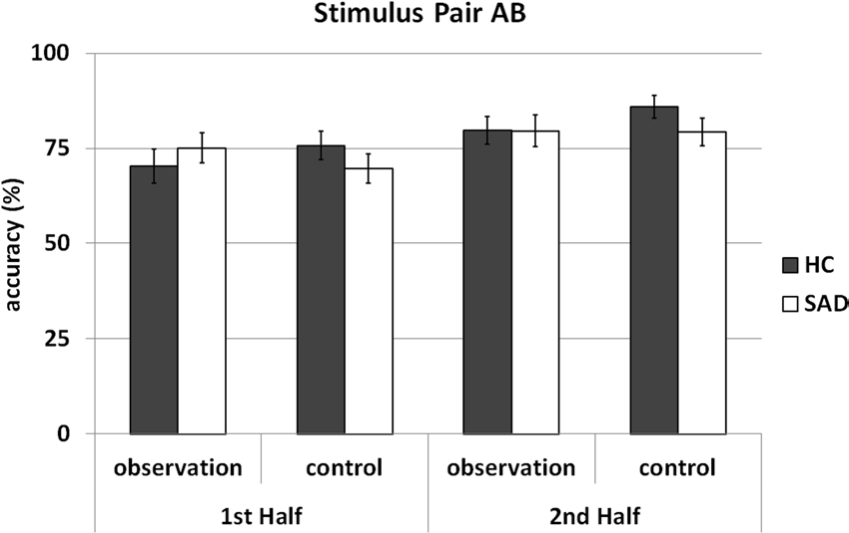
Accuracies for SAD patients and HC in the probabilistic learning task according to time (1^st^ Half and 2^nd^ Half) and condition (observation, control) for stimulus pair AB. Error bars indicate standard errors of the mean.

Figure 2 shows accuracy scores (mean percentages of correct responses) according to group (SAD/HC), time (1^st^ half, 2^nd^ half), and condition (observation/control) for stimulus pair AB. Corresponding information for stimulus pairs CD and EF is provided in the Supplementary Material.

In general, HC and SAD patients showed similar learning rates. The ANOVA yielded no significant main effect of group (F_1,62_ = 0.238, *p* = 0.627) and no interaction effects involving the group factor (all *p*s > 0.150). A significant main effect of time (F_1,62_ = 36.682, *p* < 0.001, ηp² = 0.372) emerged, indicating that accuracy was lower in the first than in the second half of each run (72.7% ± 2.24 vs. 81.12% ± 2.11, *t*(63) = −6.013, *p* < 0.001). No other main or interaction effects reached significance (all *p*s > 0.227).

**Figure 3:**
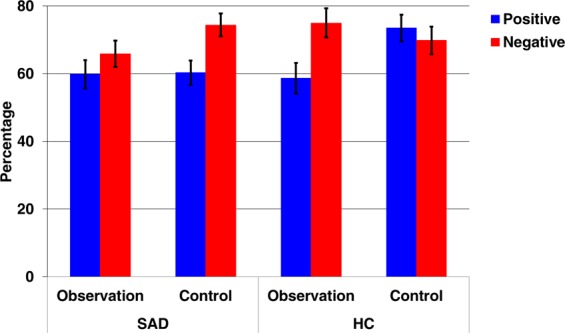
Results from the transfer learning block. Bars indicate the percentage of choosing the most positive stimulus (**A**) (blue) and avoiding the most negative stimulus (**B**) (red). Error bars indicate standard errors of the mean.

For the transfer block (for descriptive data, see Fig. 3), a significant main effect of condition was found, with an overall higher rate of correct responses in the control condition as compared to the observation condition (69.51% ± 1.99 vs. 64.85% ± 2.15; F_1,62_ = 4.207, *p* = 0.044, ηp² = 0.064). A significant main effect of valence indicated that learning from negative feedback was more pronounced than learning from positive feedback (71.28% ± 2.21 vs. 63.08% ± 2.27; F_1,62_ = 8.504, *p* = 0.005, ηp² = 0.121). These effects were further qualified by a significant condition-by-valence-by-group interaction (F_1,62_ = 7.467, *p* = 0.008, ηp² = 0.107). Post-hoc t-tests showed that HC learned better from negative as compared to positive feedback in the observation condition (*t*(29) = −2.628, *p* = 0.014), while there was no difference in the control condition (*p* = 0.475). This pattern was reversed in SAD, with patients learning better from negative feedback in the control condition (*t*(33) = −3.004, *p* = 0.005), but showing no difference in the observation condition (*p* = 0.282).

### Behavioral data – response time

**Figure 4:**
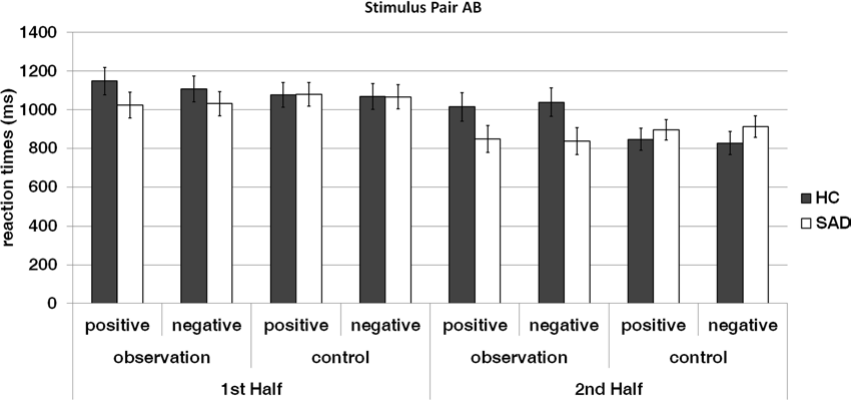
Reaction times for SAD patients and HC in the probabilistic learning task according to time (1^st^ Half and 2^nd^ Half), condition (observation, control) and feedback valence (positive, negative) for stimulus pair AB. Error bars indicate standard errors of the mean.

Figure 4 shows response times according to group (SAD/HC), time (1^st^ half, 2^nd^ half), condition (observation/control), and feedback valence (positive, negative) for stimulus pair AB. Corresponding information for the stimulus pairs CD and EF is provided in the Supplementary Material.

The repeated-measures ANOVA yielded a significant effect of time (F_1,62_ = 89.829, p < 0.001, ηp² = 0.592), with responses generally faster in the second (902.49 ms ± 37.40) as compared to the first half (1074.93 ms ± 39.89) of the experiment.

A significant interaction term emerged for the condition x group (F_1,62_ = 4.121, p = 0.047, ηp² = 0.062) interaction. This effect was further qualified by a near-significant condition-by-time-by-group (F_1,62_ = 3.911, p = 0.052, ηp² = 0.059) as well as a significant condition-by-time- by-valence- by-group (F_1,62_ = 4.040, p = 0.049, ηp² = 0.061) interaction. Post-hoc paired sample t-tests indicated that patients as compared to HC tended to show decreased response times in the second half of the observation condition when receiving negative feedback(t(62) = 1.991, p = 0.051). The other paired comparisons did not show significant differences (all *p*s > 0.108). All other main and interaction effects failed to reach significance (all *p*s > 0.125).

### (Dis)comfort ratings

Mean subjective ratings of unpleasantness and arousal level for SAD patients and HC according to feedback valence are provided in Table 2. SAD patients as compared to HC reported significantly higher arousal levels for both negative (F_1,62_ = 7.981, *p* = 0.006, ηp² = 0.114) and positive feedback (F_1,62_ = 6.431, *p* = 0.014, ηp² = 0.094) and tended to rate negative feedback as more unpleasant (F_1,62_ = 3.559, *p* = 0.064, ηp² = 0.054). In addition, patients compared to HC experienced greater discomfort during the observation condition (t(62) = −4.115, p < 0.001). No other significant main or interaction effects emerged (all *p*s > 0.121).

**Table 2:** Subjective ratings of unpleasantness and arousal level for negative and positive feedback for patients and controls in the observation and control conditions.

Variable	Controls (n = 30)		Patients (n = 34)	
*Rating*	Observation	Control	Observation	Control
	Mean (SD)	Mean (SD)	Mean (SD)	Mean (SD)
Unpleasantness of negative feedback	4,57 (±2,37)	4,47 (±2,06)	5,65 (±2,11)	5,27 (±2,29)
Unpleasantness of positive feedback	1,63 (±1,56)	1,57 (±1,28)	1,83 (±1,59)	1,44 (±,79)
Arousal level negative feedback	4,10 (±2,34)	3,50 (±2,11)	5,21 (±2,06)	5,00 (±2,16)
Arousal level positive feedback	5,20 (±2,04)	5,00 (±1,86)	6,29 (±1,51)	6,00 (±1,65)

All measures rated on a 9-point Likert scale. SD = standard deviation.

### ERP data

**Figure 5:**
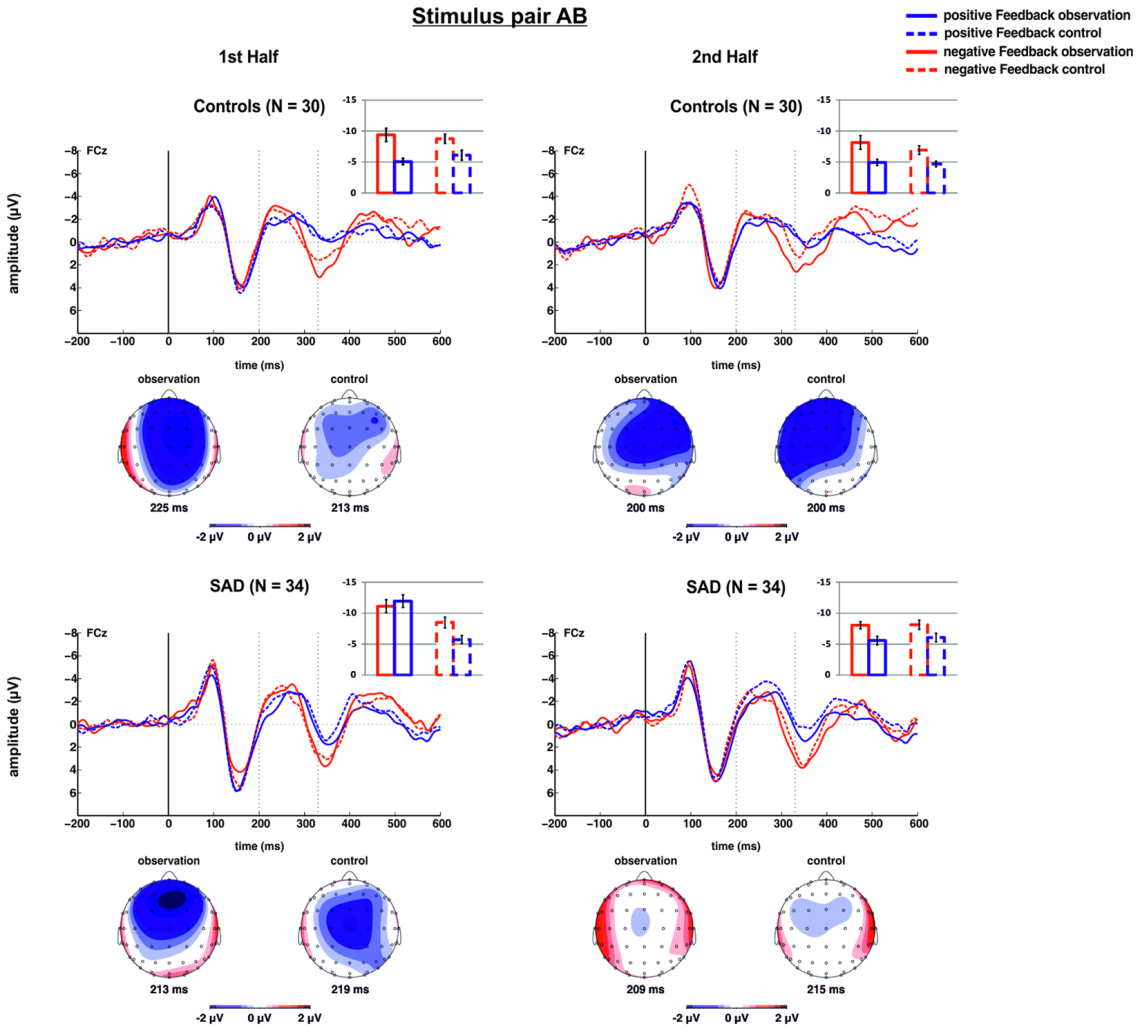
Feedback-locked original waveforms (top) and scalp topographies for post-feedback peak negativity in the difference signal (negative-positive; bottom) for stimulus pair AB at electrode FCz according to feedback valence (positive/negative) and condition (observation/control) for healthy control subjects (top) and SAD patients (bottom). Data for the first half of the run is provided on the left, for the second part of the run on the right. Bar charts represent mean FRN magnitudes resulting from the peak-to-peak analysis at electrode FCz.

**Figure 6:**
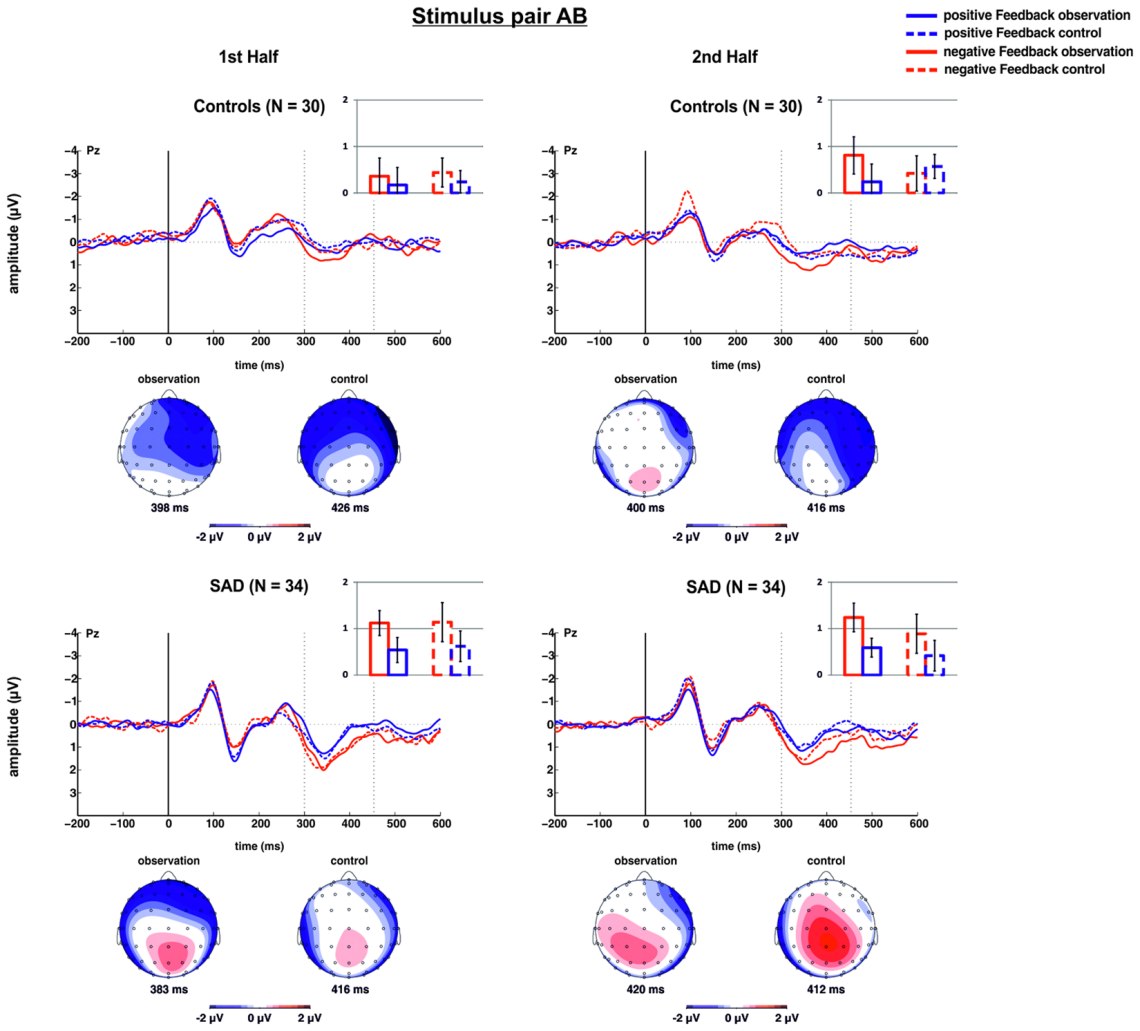
Feedback-locked original waveforms (top) and corresponding scalp topographies for the difference signal (negative-positive; bottom) for stimulus pair AB at electrode Pz for positive and negative feedback in the observation and control condition for healthy control subjects (top) and SAD patients (bottom). Data for the first half of the run are displayed on the left, for the second part of the run on the right. Bar charts represent mean P3 magnitudes in the time window 300 to 450 ms after feedback onset at electrode Pz.

Grand-average waveforms for negative and positive feedback at electrode FCz along with scalp topographies for the time points of post-feedback peak negativity in the difference signal (negative-positive) for stimulus pair AB according to condition (observation/control), time (1^st^ half/2^nd^ half), and group (SAD/HC) are provided in Fig. 5. Respective data for electrode Pz and scalp topographies for the time points of post-feedback peak positivity in the difference signal (negative-positive) are provided in Fig. 6. Please see the Supplementary Material for the corresponding figures for stimulus pairs CD and EF.

### FRN – stimulus pair AB

For stimulus pair AB, the ANOVA yielded a significant main effect of time, indicating that the FRN was larger, i.e. more negative, in the first as compared to the second half of the experiment (−8.31 µV ± 0.42 vs. −6.56 µV ± 0.35; F_1,62_ = 22.071, *p* < 0.001, ηp² = 0.263). A main effect of valence reflected that the FRN was also larger for negative as compared to positive feedback (−8.62 µV ± 0.45 vs. −6.26 µV ± 0.33; F_1,62_ = 31.053, *p* < 0.001, ηp² = 0.334). Crucially, main effects of condition (F_1,62_ = 7.269, *p* = 0.009, ηp² = 0.105) and group (F_1,62_ = 4.340, *p* = 0.041, ηp² = 0.065) indicated that the FRN was more pronounced in the observation (−8.02 µV ± 0.42) as compared to the control condition (−6.86 µV ± 0.38), and larger in SAD patients (−8.14 µV ± 0.46) as compared to HC (−6.74 µV ± 0.49), respectively. These effects were further qualified by a number of significant interaction effects that included the condition-by-group interaction (F_1,62_ = 4.263, *p* = 0.043, ηp² = 0.064), the time-by-condition interaction (F_1,62_ = 8.385, *p* = 0.005, ηp² = 0.119), the time-by-condition-by-group interaction (F_1,62_ = 18.953, *p* < 0.001, ηp² = 0.234), the time-by-valence-by-group interaction (F_1,62_ = 4.340, *p* = 0.041, ηp² = 0.065), the condition-by-valence-by-group interaction (F_1,62_ = 8.252, *p* = 0.006, ηp² = 0.117), and, finally, the time-by-condition-by-valence-by-group interaction (F_1,62_ = 5.128, *p* = 0.027, ηp² = 0.076).

Due to the high level of complexity in these results, separate repeated-measures ANOVAs were conducted for the SAD and HC groups. Results showed that in HC, only a main effect of time (F_1,29_ = 6.664, *p* < 0.015, ηp² = 0.186) and a main effect of valence (F_1,29_ = 22.849, *p* < 0.001, ηp² = 0.441) were present, with an increased FRN in the first (−7.32 µV ± 0.53) as compared to the second half (−6.17 µV ± 0.51) of the runs, and in response to negative (−8.30 µV ± 0.69) as compared to positive feedback (−5.19 µV ± 0.42). None of the remaining main effects and interactions reached statistical significance (all *p*s > 0.103). For SAD patients, main effects of time (F_1,33_ = 16.517, *p* < 0.001, ηp² = 0.334), valence (F_1,33_ = 8.555, *p* = 0.006, ηp² = 0.206), and condition (F_1,33_ = 9.503, *p* = 0.004, ηp² = 0.224) indicated that the FRN was larger in the first half of the runs (−9.32 µV ± 0.63) as compared to the second (−6.96 µV ± 0.47), for negative (−8.95 µV ± 0.59) as compared to positive feedback (−7.33 µV ± 0.50), and for the observation condition (−9.17 µV ± 0.58) as compared to the control condition (−7.11 µV ± 0.58). These effects were further qualified by interaction effects analogous to the original 2 × 2 × 2 × 2 ANOVA. These included significant interactions of time and condition (F_1,33_ = 21.476, *p* < 0.001, ηp² = 0.394), time and valence (F_1,33_ = 4.463, *p* = 0.042, ηp² = 0.119), condition and valence (F_1,33_ = 5.886, *p* = 0.021, ηp² = 0.151) and, finally, time, valence and condition (F_1,33_ = 8.320, *p* = 0.007, ηp² = 0.201).

To resolve these interactions and to further clarify the difference between groups and conditions, post-hoc t-tests were employed. The bar graphs in Fig. 5 show mean amplitudes and SEs for the different conditions. Results indicated that in patients, the FRN was increased for both negative (*t*(33) = −2.161, *p* = 0.038) and positive feedback (*t*(33) = −5.447, *p* < 0.001) in the first half of the observation condition as compared to the first half of the control condition. Furthermore, the difference between conditions (i.e., observation-control) was significantly larger for positive as compared to negative feedback in the first half of the runs (*t*(33) = 3.108, *p* = 0.004). All these effects were absent in the second half (all *p*s > 0.540), and they were exclusive to the SAD group. The respective comparisons failed to reach significance in HC (all *p*s > 0.150). HC, on the other hand, showed consistent differentiation of feedback valence across the experiment. Negative feedback was associated with stronger FRN in both the first (*t*(29) = −3.875, *p* = 0.001) and second half (*t*(29) = −3.340, *p* = 0.002) of the observation as well as the first (*t*(29) = −3.213, *p* = 0.003) and second half (*t*(29) = −2.841, *p* = 0.008) of the control condition. In SAD, the FRN was more pronounced for negative feedback in the first (*t*(33) = −3.386, *p* = 0.002) and second half (*t*(33) = −2.754, *p* = 0.002) of the control condition as well as the second half of the observation condition (*t*(33) = −3.576, *p* = 0.001). In the first half of the observation condition, however, no difference was observed for feedback valence in SAD (*p* = 0.371).

### P3 – stimulus pair AB

For the P3, the ANOVA yielded a significant main effect of valence (F_1,62_ = 9.476, *p* = 0.003, η_p_² = 0.133), with a larger P3 for negative (0.80 µV ± 0.21) as compared to positive feedback (0.42 µV ± 0.17). All other main and interaction effects failed to reach significance (all *p*s > 0.104).

### Correlations

To elucidate the relationship between learning type (positive and negative learning) and ERP measures, results from the transfer learning block were correlated with ∆FRN and ∆P3 separately for SAD and HC. To assess the influence of depressive symptoms on our data, we calculated correlations between BDI and FRN data separately for SAD and HC.

### ∆FRN

Results indicated that learning from negative feedback was negatively correlated with ∆FRN for the stimulus pair AB in SAD patients in both the first (*r* = −0.401*, p* = 0.019) and the second half (*r* = −0.379*, p* = 0.028) of the control condition. This pattern suggests that better learning from negative feedback was associated with a more pronounced FRN amplitude difference between negative and positive feedback in patients. However, this effect was not present in either half of the observation condition (*r* = −0.162*, p* = 0.359 and *r* = −0.256*, p* = 0.143, respectively). In HC, the opposite pattern emerged, with marginally significant negative correlations between learning from negative feedback and ∆FRN in the first (*r* = −0.347*, p* = 0.060) and the second half (*r* = −0.350*, p* = 0.058) of the observation condition, but not the control condition (*r* = −0.155*, p* = 0.412 and *r* = −0.275*, p* = 0.142, respectively).

For positive learning, the correlation with ∆FRN amplitudes in the second half of the experiment approached significance in SAD (*r* = −0.297*, p* = 0.088), but no further effects were found in either SAD (all *p*s > 0.288) or HC (all *p*s > 0.168).

### ∆P3

In SAD, the ∆P3 in the second half of the observation condition (*r* = 0.541*, p* = 0.002) showed a significant positive relationship with learning from positive feedback, and ∆P3 amplitudes in the first half of the control condition also tended to correlate with positive learning (*r* = 0.312*, p* = 0.094). No other coefficients reached significance in HC (all *p*s > 0.131) or SAD (all *p*s > 0.150).

### BDI

For SAD patients, significant negative correlations emerged between BDI and FRN to negative (r = −0.360, p = 0.036) as well as to positive feedback (r = −0.409, p = 0.016) in the first half of the observation condition. No significant correlations were found between FRN amplitudes and BDI in the second half of the observation condition or in the control condition (all *p*s > 0.649). In HC, no significant correlations were found (all *p*s > 0.159).

## Discussion

The objective of the current study was to examine the neural correlates of feedback processing in SAD as a function of social context, and to elucidate their relation to alterations in feedback learning. To this end, HC and SAD patients performed a probabilistic learning task under social observation, and without being observed. Patients experienced more subjective discomfort in the observation condition. Learning rates did not differ between groups or conditions. Importantly, however, SAD patients showed better learning from negative as compared to positive feedback in the control condition, while HC learned better from negative feedback in the observation condition. As expected, ERP results were most distinct for the easiest stimulus pair AB, while CD and EF showed less consistent results. Therefore, analysis focused on stimulus pair AB. Here, SAD patients demonstrated increased FRN amplitudes in the first half of the observation condition, and notably, this effect was more pronounced for positive feedback.

Results for the transfer block in the control condition are well in line with our expectations as well as theoretical frameworks of SAD^2^ and previous empirical findings^8^. In particular, they support the idea that SAD patients focus on negative rather than positive information^2,7^ and as a result learn better from negative feedback. Results in the observation condition, however, seem to contradict this idea. Here, SAD patients did not show a difference between negative and positive learning, unlike HC. ERP results may help to clarify this pattern. Correlational analyses of ∆FRN and transfer learning results show that learning from negative feedback was negatively correlated with ∆FRN in SAD patients in the control but not in the observation condition. The opposite was true for HC, mirroring results of the transfer learning blocks. Importantly, the FRN has been described as a neural marker that indexes whether a person is more prone to learn from negative or positive feedback. The present results confirm this notion, but also extend previous findings by Frank *et al*.^28^ in showing that being a negative or positive learner (and the associated differentiation in the FRN) may depend on both state- and trait-dependent characteristics. It appears that HC rely on positive reinforcement in the non-threatening control condition, but switch to a more avoidance-oriented strategy under observation, i.e. social stress. SAD patients appear to be more responsive to negative information by default. Adding stress in the form of social scrutiny, however, leads to a decline in learning from negative feedback, accompanied by a lack of differentiation in FRN amplitudes. The classic social facilitation theory^38^ holds that social observation leads to better performance on easy, well-learned tasks, but impairs performance on novel or difficult tasks. In addition, an inverted u-shaped function between arousal and performance has been proposed, so that ideal performance emerges at medium levels of arousal and/or anxiety^39,40^. In SAD patients, who reported generally higher levels of arousal and discomfort during observation, arousal may have exceeded the level of optimal performance in the observation condition. It should be noted, however, that performance in the learning phase was not generally impaired in SAD.

Aside from their relationship with behavioral findings, the ERP results showed a complex pattern, with differences between groups and conditions modulated by several factors. The most interesting results were found for the AB stimulus pair, i.e. the stimulus pair with the highest action feedback contingency. The FRN was more pronounced after negative as compared to positive feedback and in the first as compared to the second half of the runs. It was also larger in the observation condition than in the control condition, and amplitudes were increased in SAD patients relative to HC. All these factors also showed significant interactions with one another. Most importantly, the FRN was significantly increased in SAD patients in the first half of the observation condition, and this effect was more pronounced for positive feedback. These results contradict the Clark and Wells^2^ model, as they point to increased (rather than decreased) processing of external information under social threat. They are well in line with the predictions of the model proposed by Rapee and Heimberg^3^, however, namely that external information may be increasingly attended to under social threat. As previous research has found internal markers of performance monitoring (namely the ERN) to be augmented in SAD as well^4,41^, the present results add to the idea that both internal and external monitoring processes may be increased in SAD^30^.

Concerning the valence-dependent effect, our results are in line with findings reported by Cao *et al*.^26^ who found that SAD patients showed a larger FRN to positive as compared to negative social feedback. While the two studies differ in important methodological aspects, both seem to suggest that the processing of positive feedback is especially altered in SAD. Cao *et al*.^26^ proposed that this might be either due to the patients’ expectancy to be socially rejected, rendering positive feedback an especially unexpected event, or due to impaired processing of negative feedback. Support for the former notion was provided in an fMRI study in which high socially anxious individuals showed increased activations in ventromedial prefrontal cortex and ACC in response to positive compared to negative and to social compared to computer feedback^42^. Moreover, the results of the present study showed that the FRN to negative feedback was more pronounced in SAD in the control condition, arguing against the notion of generally impaired processing of negative feedback in SAD^26^. The reduced valence-dependent differentiation of the FRN in the observation condition might also be attributable to reduced attention to feedback information^2^. We are inclined to think that reduced attention to feedback in general should be associated with decreased learning performance (which was not observed in SAD in the present study), but we did not specifically assess attention to feedback (e.g., by quantifying or characterizing eye movements while feedback stimuli were presented). This would be an interesting aspect to explore in future studies.

As recent studies have shown that the neural response to positive rather than negative feedback may account for most of the variation in the FRN^15,17^, an alternative explanation might also be considered. Following the idea that the FRN results from a reward positivity modulating an N2-like component^14–16^, this would imply that the patients’ increased FRN to positive feedback in the observation condition reflects decreased processing of positive feedback. This would align with other studies reporting altered processing of positive feedback in SAD. Boehme *et al*.^43^, for instance, found decreased activation of the ventral striatum in SAD patients compared to HC during anticipation of giving a speech. Becker *et al*.^27^ reported that SAD patients compared to HC failed to exhibit increased activity in reward-associated areas after positive feedback under social observation. In previous EEG studies, temporo-spatial principal component analysis (PCA) was used to disentangle the different sub-processes contributing to the FRN^16,44^. In an exploratory analysis we were unable to identify a clear reward-positivity in our data by means of temporo-spatial PCA, raising doubts as to whether reduced reward-related processing can account for the findings of the present study in SAD. Further research is needed to investigate how FRN sub-processes may be altered in SAD.

In any case, these findings might suggest that under social observation, SAD patients do not process positive information adequately on the neural level, possibly leading to blurred differentiation of negative and positive reinforcement and potentially contributing to the collapse of learning from negative feedback. In this respect, the present findings may also have implications for treatment of SAD in psychotherapy. Depending on patients’ specific deficits, it may be helpful to focus more on strengthening the ability to distinguish feedback valence or on the ability to adequately encode and retrieve feedback.

With regard to the varying reward contingencies of the current design, we found group differences only for stimulus pair AB, whereas the other two stimulus pairs largely failed to show effects. This might indicate that differences between groups emerge only when actual learning occurs and feedback is informative. While most participants performed above chance on the AB trials (which were the easiest to learn), this number dropped substantially for the CD and EF trials. Along these lines, it is conceivable that many participants did not actually learn the underlying contingencies and feedback thus was perceived as random and non-informative. Taking into account the effects of expectancy and surprise on FRN amplitude^20,45–47^, it is conceivable that potential differences between groups and conditions were blurred due to the higher level of variance in the CD and EF trials. This assumption is corroborated by the fact that group differences for the AB pair were present in the first half, but declined in the second half of the observation condition. To the best of our knowledge, the present study is the first to demonstrate that electrophysiological correlates of feedback learning are not only altered in SAD, but that this alteration changes over time. On the one hand, this may be ascribed to habituation effects, with patients becoming gradually accustomed to the social scrutiny manipulation. Previous research has shown that while habituation of peripheral physiological parameters such as heart rate is mostly comparable between high and low socially anxious individuals^48–50^, habituation of neural activity in a wide range of brain regions may be significantly affected by social fearfulness^51,52^. The present study may add to these results by showing that SAD patients’ performance monitoring system is hyper-vigilant at the start of the observation condition, but normalizes over the course of the run.

On the other hand, alterations of the FRN in SAD may also be explained in the context of learning as a dynamic process. Feedback is most informative at the beginning of the run, when action-outcome contingencies are still unknown. At this stage, the performance monitoring system relies on external feedback, reflected in distinct coding of positive and negative feedback in the FRN, whereas the response-locked error-related negativity (ERN)^53,54^ and the correct-related negativity (CRN) do not differentiate between correct and incorrect responses^55,56^. Over the course of the learning process, contingencies are increasingly internalized. Differential coding of positive and negative feedback in the FRN is decreased, while ERN and CRN amplitudes now clearly code incorrect and correct responses^55,56^. The present results might suggest that the neural dynamics of this process are altered in SAD. This notion is corroborated by results obtained by Judah *et al*.^57^, who reported that differences between high and low socially anxious individuals in FRN amplitude were present only before learning of stimulus-response mappings had occurred. Several other studies have shown that social anxiety is associated with ERN alterations^4,41,57,58^. Along these lines, it is conceivable that an increased FRN is present only during the initial learning of stimulus-response mappings in SAD, but that persistent hyperactivity of the performance monitoring system is reflected in ERN enhancement after learning. Further research involving more specifically designed task procedures is needed to test this hypothesis.

While accuracy was comparable between groups, differences in response times appear to complement differences in ERP data to some degree. In particular, one might expect SAD patients to show a more pronounced speed-accuracy trade-off in the sense that their response times might be prolonged compared to HC due to a more cautious response style, especially in the observation condition. However, the results showed that SAD patients did not reduce response speed during the observation condition, while HC did. More specifically, patients tended to show decreased response times in the second half of the observation condition for choices associated with negative feedback. This result is quite the opposite of what one might expect based on theoretical assumptions. In fact, it appears that HC were more cautious under social observation, while SAD patients employed a response strategy that was faster, i.e. more risk-prone, once contingencies had been learned. While we certainly would have expected SAD patients to be more risk-aversive, this result might still be explained in terms of SAD pathology. In particular, it is reminiscent of SAD patients’ tendency to ‘speed up’ during speeches, which can be attributed to SAD patients applying exaggerated standards to their own performance^3^.

When interpreting the results of the present study, some limitations need to be taken into account. First, it should be noted that the operationalization of the social context manipulation did not involve a full distinction between an experimental social and a non-social control condition. In particular, the experimenter was still present in the same room in the control condition, (although eye contact was prevented) so that differences between the two conditions may have been blurred. We would expect observation effects as found in the present study to be augmented with even more rigorous manipulations of social context. Furthermore, since SAD patients showed a significantly higher level of depressiveness as indicated by BDI scores, effects of depressiveness may have influenced our results. We found negative correlations between FRN and BDI for negative and positive feedback in the first half of the observation condition in SAD patients, but not in the second half and not in the control condition. In HC, no significant correlations emerged at all. In general, SAD patients tend to show increased levels of depressiveness^59,60^, making it hard to disentangle the specific contributions of anxiety and depressiveness to neurophysiological data. Empirical findings regarding the link between depression and FRN amplitudes are quite inconsistent. Tucker *et al*.^61^ found larger FRNs, particularly following negative feedback, in moderately depressed patients, and Santesso *et al*.^62^ confirmed this pattern in subjects with remitted depression. In contrast, a very recent meta-analysis^63^ reported that FRN amplitudes were blunted in depressed patients. In our study, the negative correlation between FRN and BDI in the SAD sample suggests that amplitudes were more negative, i.e. the FRN was larger, when BDI scores were higher, and this was true for both positive and negative feedback, thus aligning with the findings by Tucker *et al*.^61^ and Santesso *et al*.^62^ However, FRN amplitudes were not correlated with BDI scores in the control condition, which speaks against a profound effect of depressiveness on FRN amplitudes in the current study.

Finally, as some of the SAD patients received antidepressive treatment, effects of medication need to be considered. However, this concerned relatively few subjects, and previous work has suggested that SSRIs do not affect ERP measures of performance monitoring directly^64,65^ (but also see^66^).

In conclusion, the present results demonstrate that feedback processing in SAD is altered as a function of several factors including, most prominently, social context. In particular, learning from negative feedback was increased in SAD patients, but this effect disappeared under social observation, while HC showed a reverse pattern. Simultaneously, a lack of differentiation between positive and negative feedback was found in SAD patients in FRN amplitudes in the observation condition during initial learning of reward contingencies for the stimulus pair with the highest action-reward contingency. Importantly, correlational analyses suggested that this absence of differentiation might explain the patients’ decreased ability to learn from negative feedback in the observation condition. Thus, the present results show altered feedback processing in SAD in the initial phase of social scrutiny, with impaired ability to differentiate between negative and positive outcomes.

## Supplementary information

Supplementary Information
