# Supplementary material for: Modulation of feedback processing by social context in social anxiety disorder (SAD)–an event-related potentials (ERPs) study
Source: Sci Rep. 2019 Mar 18;9:4795. doi: 10.1038/s41598-019-41268-0 (PMC6423138; doi:10.1038/s41598-019-41268-0)
Supplement: Supplementary file 1 — Supplementary Information [file 41598_2019_41268_MOESM1_ESM.docx]

**Modulation of feedback processing by social context in social anxiety disorder (SAD)–**

**an event-related potentials (ERPs) study**

***Supplementary Information***

Rolf Voegler^1*^, Jutta Peterburs^1,2*^, Christian Bellebaum^2^ & Thomas Straube^1^

^1^Institute of Medical Psychology and Systems Neuroscience, University of Münster, Von-Esmarch-Str. 52, 48149 Münster, Germany

^2^Department of Biological Psychology, Institute for Experimental Psychology, Heinrich-Heine-University, Universitätsstraße 1, 40225 Düsseldorf, Germany

Address for correspondence:

Rolf Voegler

Institute of Medical Psychology and Systems Neurosciences

University of Muenster, Germany

Phone: +49 251 83 57174

Fax: +49 251 83 55494

Email: [rolf.voegler@uni-muenster.de](mailto:rolf.voegler@uni-muenster.de)

***Results***

***Behavioral data - accuracies***

Figure 7 shows accuracy scores (mean percentages of correct responses) according to group (SAD/HC), time (1^st^ half, 2^nd^ half), and condition (observation/control) for stimulus pair CD. HC and SAD patients showed similar learning rates. The repeated-measures ANOVA yielded no significant main effect of group (F_1, 62_ = .165, *p =* .686) and no interaction effects involving the group factor (all *p*s>.225). A significant main effect of time (F_1, 62_ = 21.448, *p <* .001, ηp² = .257) emerged, indicating higher accuracy in the second half of each run (66.30% ± 1.99 vs. 73.15% ± 2.23). No other main or interaction effects reached significance (all *p*s > .272).

***
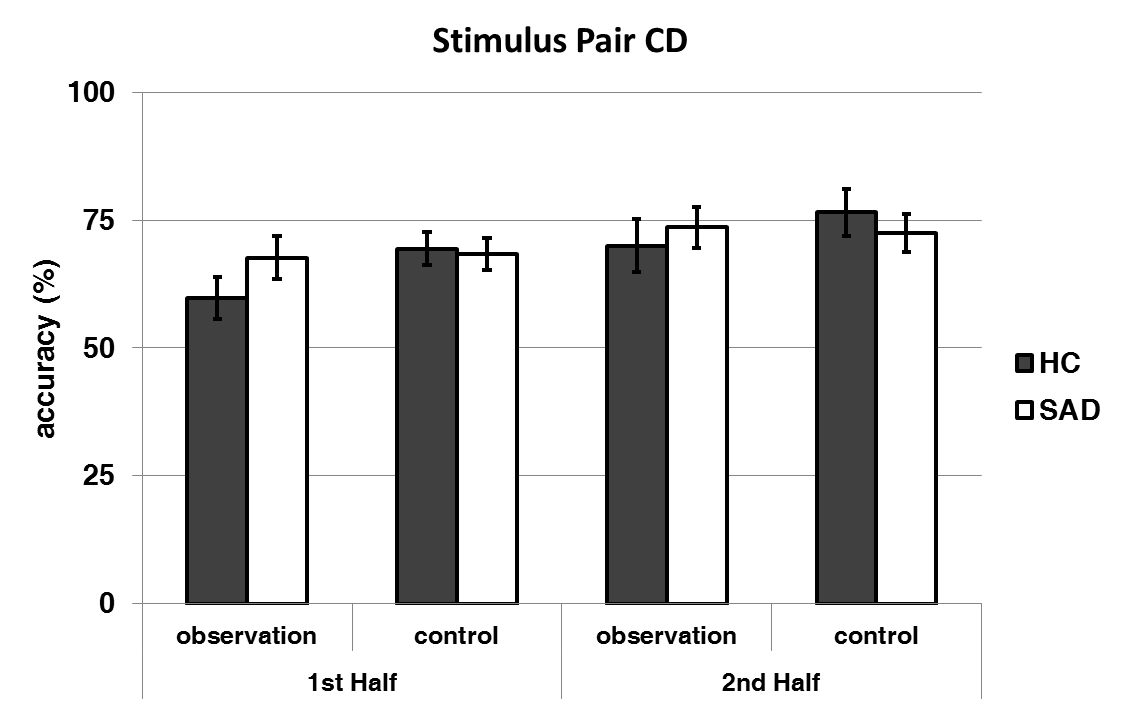
***

Figure 7: Accuracies for SAD patients and HC in the probabilistic learning task according to time (1^st^ Half and 2^nd^ Half) and condition (observation, control) for stimulus pair CD. Error bars indicate standard errors of the mean.

Figure 8 shows accuracy scores (mean percentages of correct responses) according to group (SAD/HC), time (1^st^ half, 2^nd^ half), and condition (observation/control) for stimulus pair EF. HC and SAD patients showed similar learning rates. The repeated-measures ANOVA yielded no significant main effect of group (F_1, 62_ = .012, *p =* .912) and no interaction effects involving the group factor (all *p*s>.459). A significant main effect of condition emerged, indicating that accuracy was higher in the control as compared to the observation condition (71.7% ± 2.40 vs. 67.75% ± 2.92, F_1, 62_ = 11.934, *p <* .001, ηp² = .161). No other main or interaction effects reached significance (all *p*s > .289).


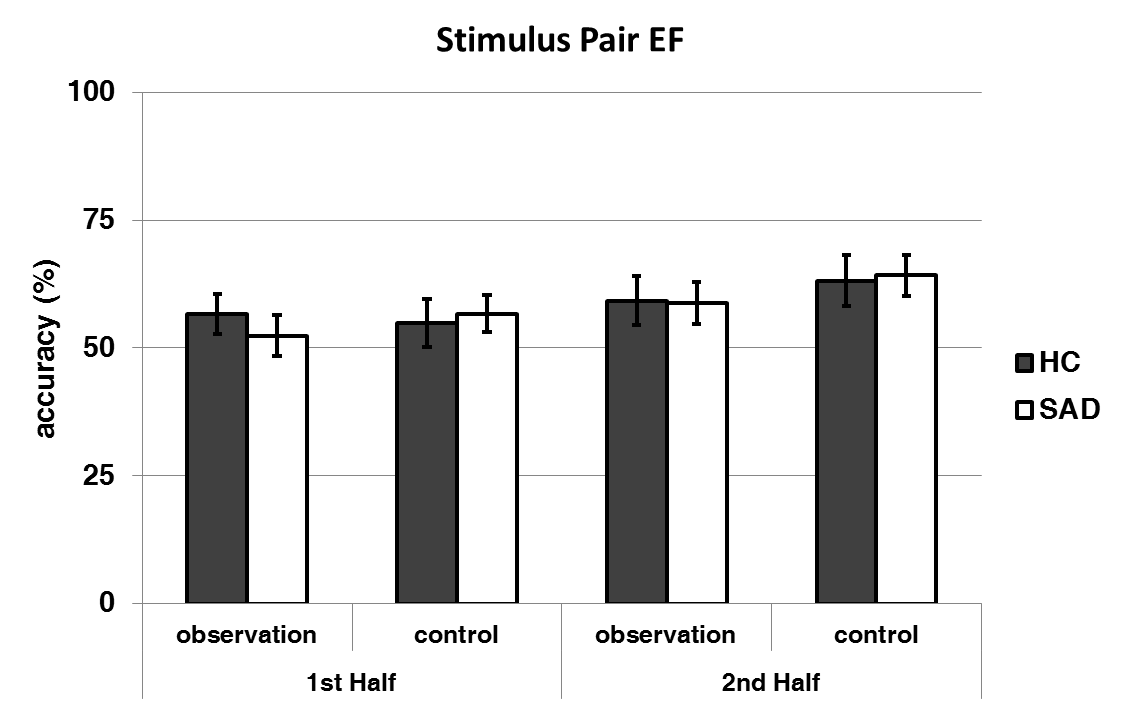


Figure 8: Accuracies for SAD patients and HC in the probabilistic learning task according to time (1^st^ Half and 2^nd^ Half) and condition (observation, control) for stimulus pair EF. Error bars indicate standard errors of the mean.

***Behavioral data – reaction times***

Figure 9 shows reaction times according to group (SAD/HC), time (1^st^ half, 2^nd^ half), condition (observation/control) and feedback valence (positive, negative) for stimulus pair CD. The repeated-measures ANOVA yielded a significant effect of time (F_1, 62_ = 140.484, p < .001, ηp² = .694), with responses being generally faster in the second (946.08ms ± 33.95) as compared to the first half (1149.90ms ± 38.03) of the experiment.

The condition x group showed a trend towards significance (F_1, 62_ = 3.294, p = .074, ηp² = .50), all other main and interaction effects were non-significant (all *p*s > .132).

***
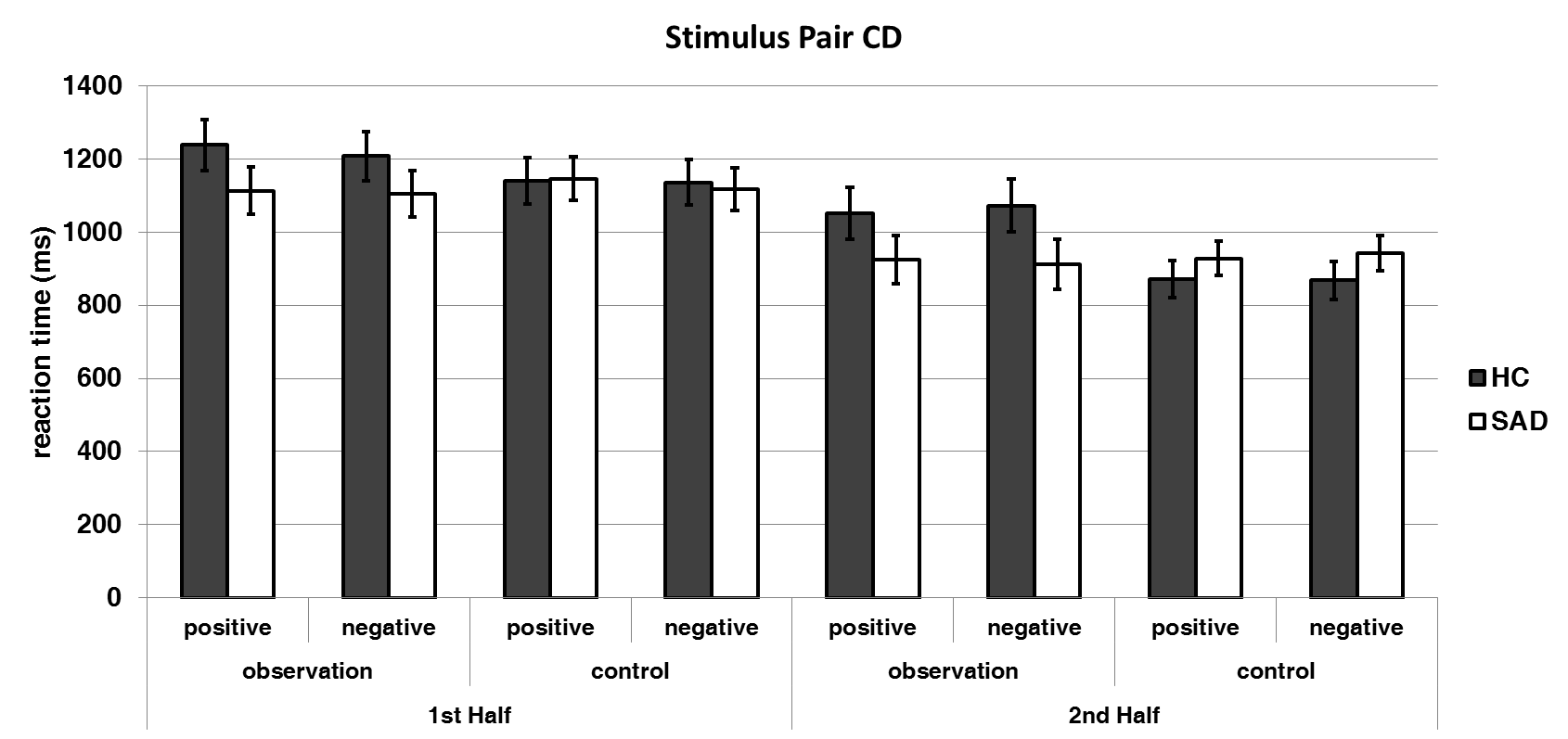
***

**Figure 9: Reaction times for SAD patients and HC in the probabilistic learning task according to time (1^st^ Half and 2^nd^ Half), condition (observation, control) and valence (correct, incorrect) for stimulus pair CD. Error bars indicate standard errors of the mean.**

Figure 10 shows reaction times according to group (SAD/HC), time (1^st^ half, 2^nd^ half), condition (observation/control) and feedback valence (positive, negative) for stimulus pair EF. The repeated-measures ANOVA yielded a significant effect of time (F_1, 62_ = 56.532, p < .001, ηp² = .477), with responses being generally faster in the second (1000.71ms ± 38.02) as compared to the first half (1153.91ms ± 36.20) of the experiment.

The time x valence x group interaction showed a trend towards significance (F_1, 62_ = 3.595, p = .063, ηp² = .55), all other main and interaction effects were non-significant (all *p*s > .139).

***
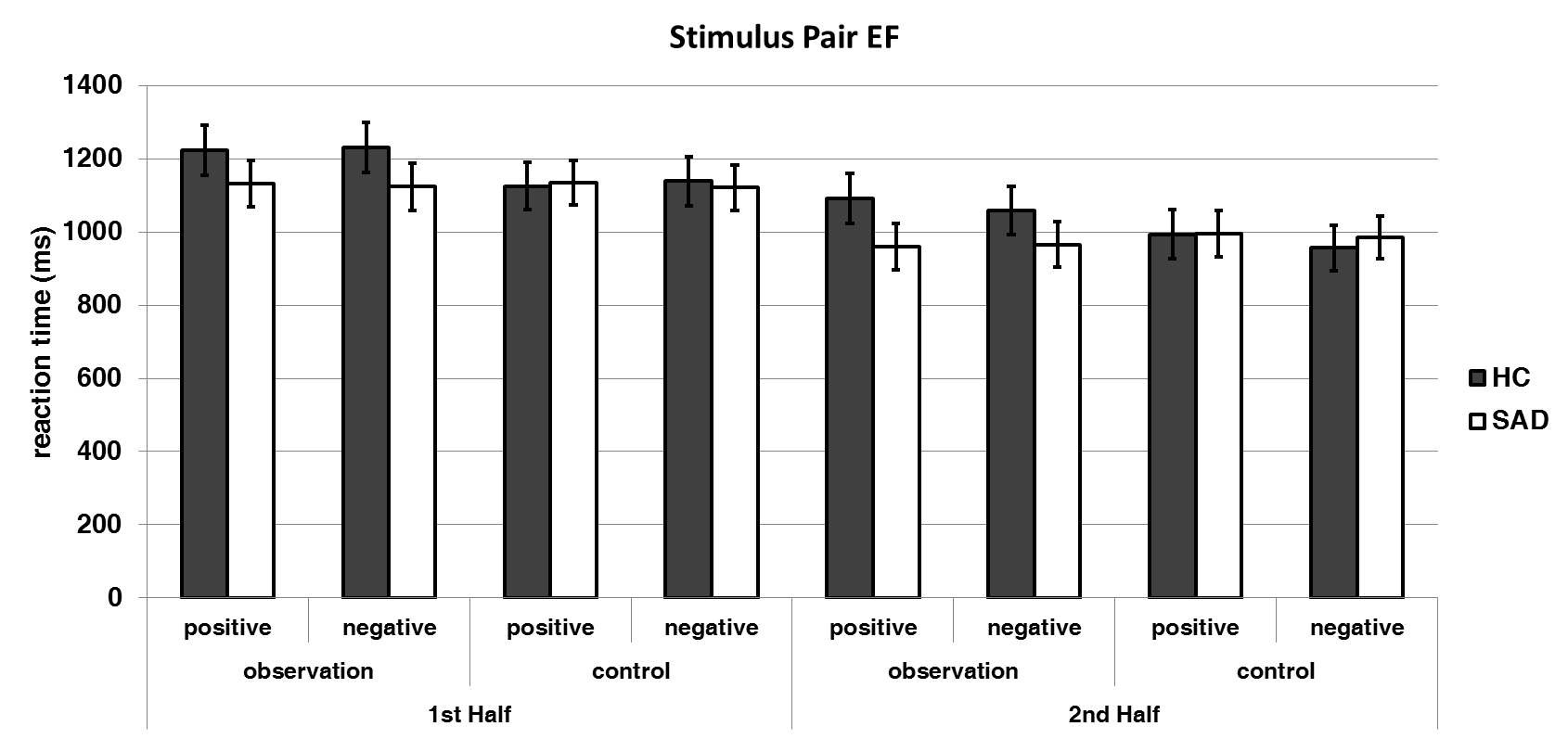
***

**Figure 10: Reaction times for SAD patients and HC in the probabilistic learning task according to time (1^st^ Half and 2^nd^ Half), condition (observation, control) and valence (correct, incorrect) for stimulus pair EF. Error bars indicate standard errors of the mean.**

**ERP data**

FRN – stimulus pair CD

For symbol pair CD, the ANOVA yielded a significant main effect of valence (F_1, 62_ = 22.923, *p <* .001, ηp² = .270), indicating that the FRN was larger for negative (-7.56µV ± .37) as compared to positive feedback (-6.00µV ± .39). The interaction of condition and valence approached significance (F_1, 62_ = 3.231, *p =* .077, ηp² = .05), but all other main and interaction effects failed to reach significance (all *p*s > .142).

FRN – stimulus pair EF

For symbol pair EF, the ANOVA yielded a significant main effect of valence (F_1, 62_ = 15.399, *p <* .001, ηp² = .199), with FRN amplitudes again larger for negative (-7.15µV ± .37) as compared to positive feedback (-5.99µV ± .34). All other main and interaction effects failed to reach significance (all *p*s > .142).

P3 – stimulus pair CD

Again, a main effect of valence was found (F_1, 62_ = 10.147, *p* = .002, η_p_² = .141), reflecting a larger P3 for negative (.71µV ± .19) as compared to positive feedback (.43µV ± .17). All other main and interaction effects failed to reach significance (all *p*s > .146).

P3 – stimulus pair EF

No main or interaction effects were found for P3 for stimulus pair EF (all *p*s > .111)

**
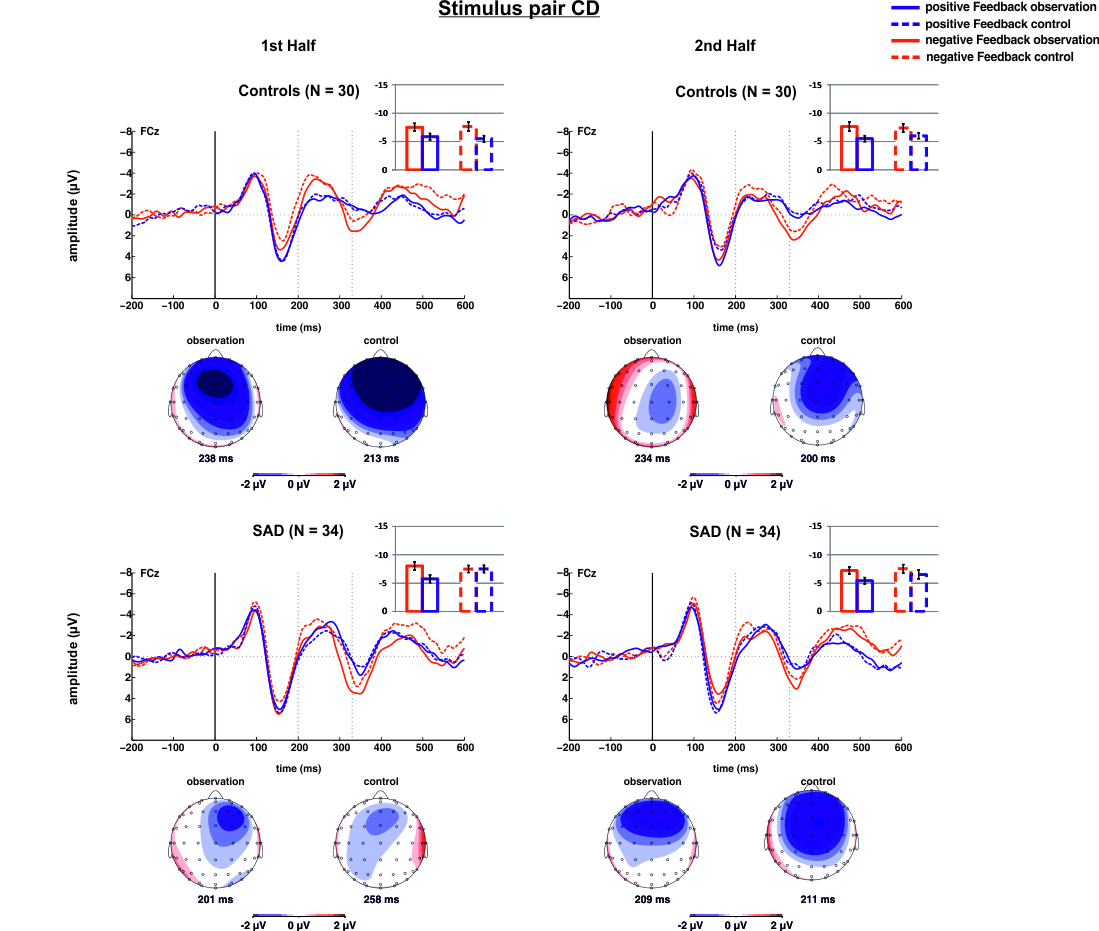
**

Figure 11: Feedback-locked original waveforms (top) and scalp topographies for post-feedback peak negativity in the difference signal (negative-positive; bottom) for stimulus pair CD at electrode FCz according to feedback valence (positive/negative) and condition (observation/control) for healthy control subjects (top) and SAD patients (bottom). Data for the first half of the run is provided on the left, for the second part of the run on the right. Bar charts represent mean FRN magnitudes resulting from the peak-to-peak analysis at electrode FCz.


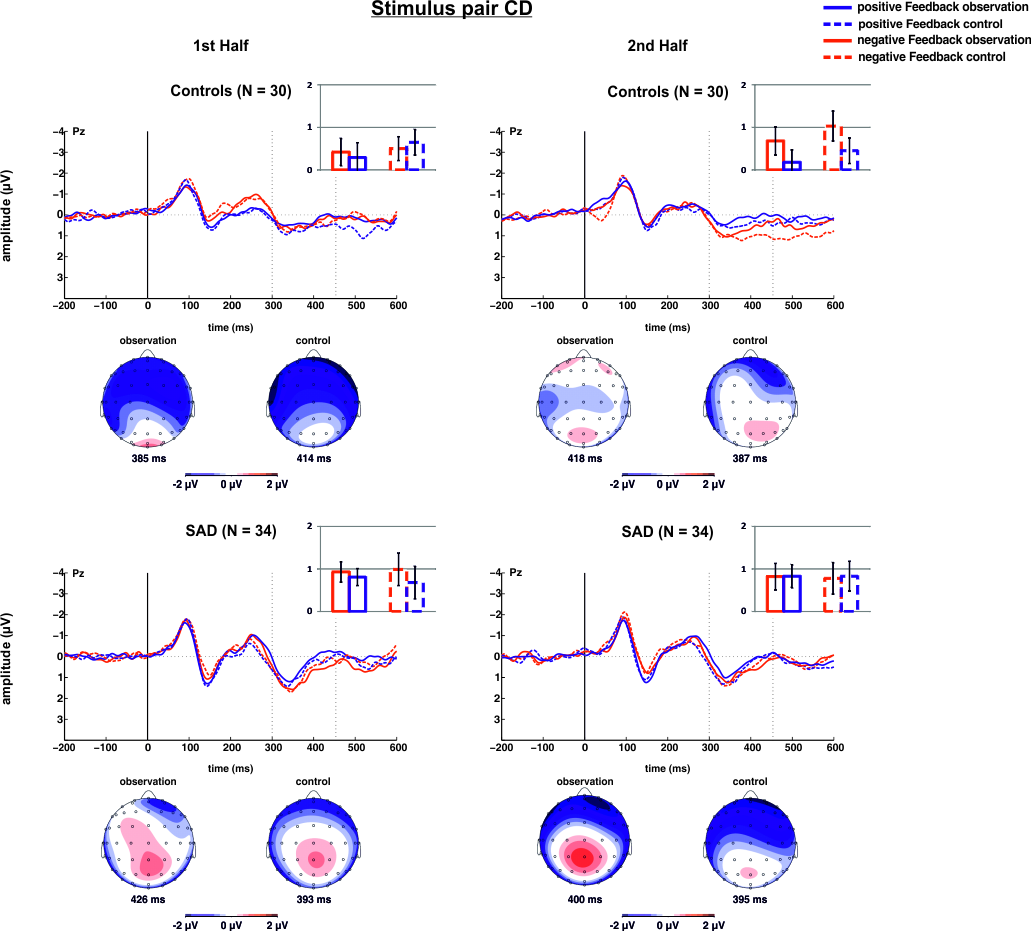


Figure 12: Feedback-locked original waveforms (top) and corresponding scalp topographies for the difference signal (negative-positive; bottom) for stimulus pair CD at electrode Pz for positive and negative feedback in the observation and control condition for healthy control subjects (top) and SAD patients (bottom). Data for the first half of the run are displayed on the left, for the second part of the run on the right. Bar charts represent mean P3 magnitudes in the time window 300 to 450ms after feedback onset at electrode Pz.


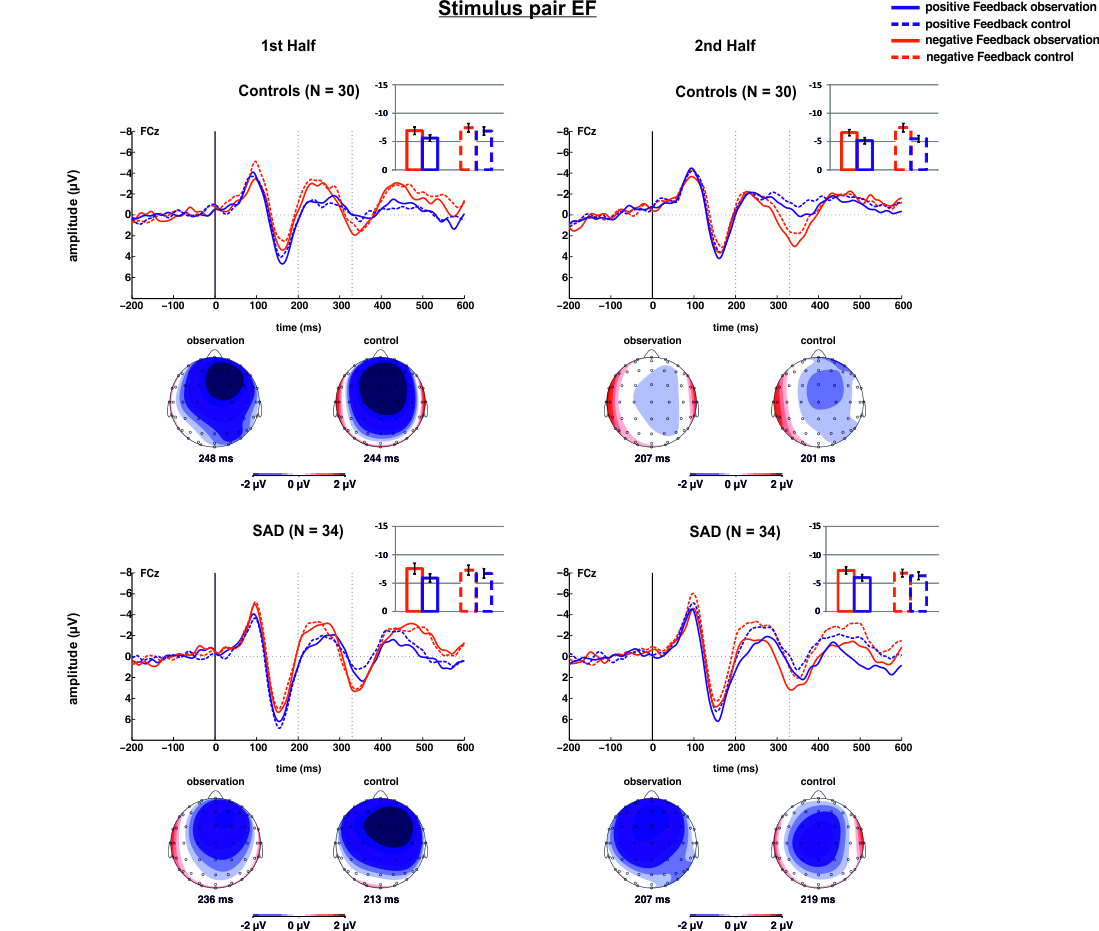


Figure 13: Feedback-locked original waveforms (top) and scalp topographies for post-feedback peak negativity in the difference signal (negative-positive; bottom) for stimulus pair EF at electrode FCz according to feedback valence (positive/negative) and condition (observation/control) for healthy control subjects (top) and SAD patients (bottom). Data for the first half of the run is provided on the left, for the second part of the run on the right. Bar charts represent mean FRN magnitudes resulting from the peak-to-peak analysis at electrode FCz.


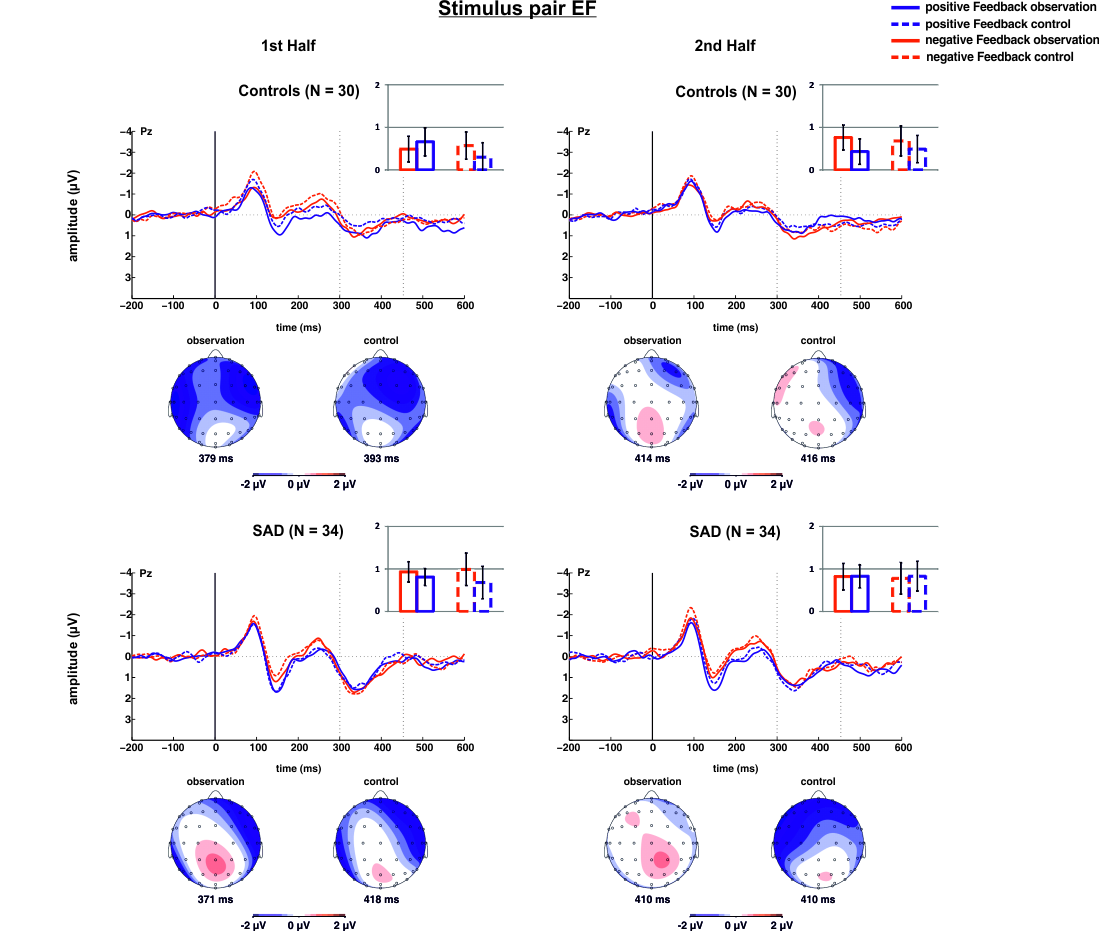


Figure 14: Feedback-locked original waveforms (top) and corresponding scalp topographies for the difference signal (negative-positive; bottom) for stimulus pair EF at electrode Pz for positive and negative feedback in the observation and control condition for healthy control subjects (top) and SAD patients (bottom). Data for the first half of the run are displayed on the left, for the second part of the run on the right. Bar charts represent mean P3 magnitudes in the time window 300 to 450ms after feedback onset at electrode Pz.
